# Creatine Supplementation Beyond Athletics: Benefits of Different Types of Creatine for Women, Vegans, and Clinical Populations—A Narrative Review

**DOI:** 10.3390/nu17010095

**Published:** 2024-12-29

**Authors:** Jorge Gutiérrez-Hellín, Juan Del Coso, Arturo Franco-Andrés, José M. Gamonales, Mário C. Espada, Jaime González-García, Miguel López-Moreno, David Varillas-Delgado

**Affiliations:** 1 Exercise and Sport Science, Faculty of Health Sciences, Universidad Francisco de Vitoria, 28223 Pozuelo, Spain; arturo.franco@ufv.es (A.F.-A.); martingamonales@unex.es (J.M.G.); jaime.gonzalez@ufv.es (J.G.-G.); david.varillas@ufv.es (D.V.-D.); 2 Sport Sciences Research Centre, Rey Juan Carlos University, 28943 Fuenlabrada, Spain; 3 Training Optimization and Sports Performance Research Group (GOERD), Faculty of Sport Science, University of Extremadura, 10005 Cáceres, Spain; 4 Facultad de Educación y Psicología, Universidad de Extremadura, 06006 Badajoz, Spain; 5 Instituto Politécnico de Setúbal, Escola Superior de Educação, 2914-504 Setúbal, Portugal; mario.espada@ese.ips.pt; 6 Life Quality Research Centre (CIEQV), Complexo Andaluz, 2040-413 Rio Maior, Portugal; 7 Faculdade de Motricidade Humana (CIPER), Universidade de Lisboa, 1499-002 Cruz Quebrada, Portugal; 8 Sport Physical Activity and Health Research & INnovation CenTer (SPRINT), 2040-413 Rio Maior, Portugal; 9 Comprehensive Health Research Centre (CHRC), University of Évora, Largo dos Colegiais 2, 7000-645 Évora, Portugal; 10 Diet, Planetary Health and Performance, Faculty of Health Sciences, Universidad Francisco de Vitoria, 28223 Pozuelo, Spain; miguel.lopez@ufv.es

**Keywords:** creatine kinase, dietary supplementation, energy metabolism, plant-based diets, exercise performance

## Abstract

Creatine monohydrate supplementation is widely used by athletes in high-intensity, power-based sports due to its ability to enhance short-term performance by increasing intramuscular phosphocreatine (PCr) stores, which aid in ATP resynthesis during intense muscle contractions. However, emerging evidence suggests that creatine monohydrate offers benefits beyond athletic performance. This narrative review explores the literature supporting the advantages of creatine supplementation in women, vegans, and clinical populations. In women, who typically have lower baseline intramuscular creatine levels, supplementation may help alleviate fatigue-related symptoms associated with the menstrual cycle, particularly during the early follicular and luteal phases. For vegans and vegetarians, who often have reduced creatine stores due to the absence of creatine-rich animal products in their diet, supplementation can improve both physical and cognitive performance while supporting adherence to plant-based diets. Additionally, creatine supplementation holds potential for various clinical populations. It may mitigate muscle wasting in conditions such as sarcopenia and cachexia, support neuroprotection in neurodegenerative diseases such as Parkinson’s and Huntington’s, improve exercise capacity in cardiovascular diseases, and enhance energy metabolism in chronic fatigue syndrome. Creatine may also aid recovery from traumatic brain injury by promoting brain energy metabolism and reducing neuronal damage. In conclusion, creatine monohydrate supplementation can enhance physical performance, cognitive function, and overall health in women, vegans, and clinical populations by addressing creatine deficiencies, improving energy metabolism, and supporting recovery from physical and neurological challenges. Most available evidence supports the effectiveness of creatine monohydrate, which should be considered the preferred form of creatine supplementation over other variants. Additionally, proper creatine dosing is essential to maximize benefits and minimize potential adverse effects that may arise from chronic ingestion of excessively high doses.

## 1. Creatine

**Figure 1:**
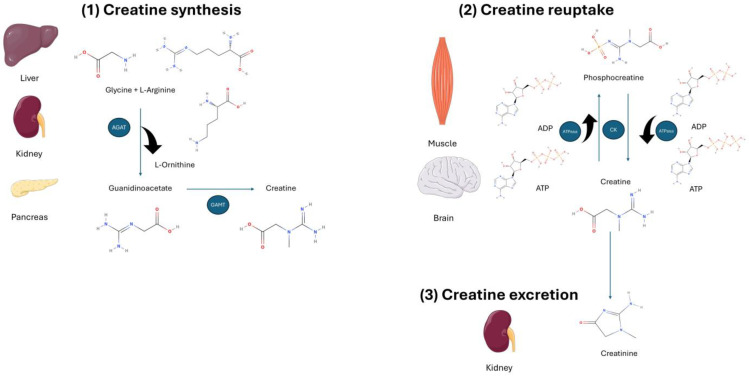
Creatine synthesis, reuptake, and excretion. (**1**) Creatine is synthesized from L-arginine and glycine in the liver, kidney, and pancreas with the help of L-arginine:glycine amidinotransferase (AGAT) in a first step, and through guanidinoacetate N-methyltransferase (GAMT) in a second step. (**2**) Creatine is released into the circulation and transported to varying tissues such as skeletal muscle and the brain. Once in the cell, creatine can be transformed into phosphocreatine by creatine kinase (CK). The high-energy stores of phosphocreatine can be used within the cell for ATP-dependent processes via ATPase enzymes. Both creatine and phosphocreatine are naturally metabolized into creatinine via a non-enzymatic reaction. (**3**) Creatinine diffuses freely into the circulation to be transported to the kidney, and it is finally excreted in the urine.

Creatine (C_4_H_9_N_3_O_2_) is a ubiquitous molecule synthesized predominantly in the liver, kidneys, and pancreas at a rate of ~1 g/day via an interorgan process. Creatine is produced naturally in two steps from the amino acids glycine and arginine [1]. These reactions are catalyzed by the enzymes L-glycine-arginine amidinotransferase (AGAT) and guanidinoacetate methyltransferase (GAMT; Figure 1) [2]. Once synthesized, creatine is transported through the bloodstream to its target tissues via sodium- and chloride-dependent creatine transporters [3]. Most of the body’s pool of creatine is in tissues with high energy demands, such as the muscle and brain, where it plays a critical role in recycling adenosine triphosphate (ATP). Recycling of ATP is achieved by converting adenosine diphosphate (ADP) back to ATP via the donation of phosphate groups. Creatine itself can be phosphorylated by creatine kinase to phosphocreatine (PCr), which is used as an energy store in skeletal muscles and the brain. The critical role of creatine in rapid energy production results in approximately 95% of the body’s creatine being stored in skeletal muscle [4]. Within the muscle, approximately 40% of creatine is in the free form and the remaining 60% is in the phosphorylated form as PCr [5]. Significant levels of creatine have also been found in other cells of the body, such as neurons, cardiomyocytes, and hepatocytes [6,7]. In addition to its role in energy production, creatine also plays a crucial role in buffering within various tissues, which expands the importance of this substance for situations with extraordinary energy demands.

The final product of creatine metabolism is creatinine, formed by the non-enzymatic conversion of phosphocreatine and creatine [8,9]. Creatinine is excreted by the kidneys at around 1 g/day and is considered a marker of glomerular filtration rate to detect possible renal pathology [9]. Because skeletal muscle contains most of the body’s phosphocreatine and creatine reserve, urinary creatinine excretion varies according to muscle mass, being on average lower in women than in men [10].

**Figure 2:**
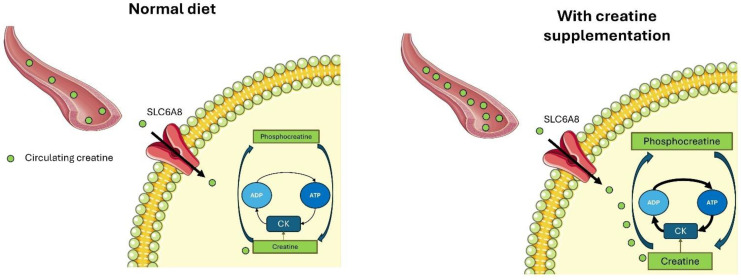
Graphical representation of the amount of creatine present within the blood circulation and within tissues through foods (normal diet) or with the use of dietary supplements of creatine (with creatine supplementation). The ingestion of dietary supplements with creatine produces higher serum creatine concentrations than the use of foods. Cellular uptake of creatine from the circulation is mediated by a creatine transporter known as SLC6A8. A higher serum creatine concentration implies a higher saturation of creatine within muscles, which in turn allows higher rates of ATP resynthesis.

In addition to endogenous synthesis, creatine can also be obtained exogenously through the consumption of certain foods. This ensures that the total body store of creatine is maintained, as creatine used in body tissues can be replenished from the diet or de novo synthesis [11]. Creatine can be ingested through foods such as meat and fish [12,13]. A normal diet contains 1–2 g/day of creatine and allows muscle stores to be saturated at 60–80%. However, this amount can significantly vary depending on the total energy intake and the type of foods ingested. Overall, dietary creatine intake serves to increase muscle creatine and PCr stores by 20–40% [14]. While creatine is naturally present in animal-based foods, dietary supplements offer a more practical, efficient, and reliable means of increasing creatine intake. This is because dietary supplements of creatine provide higher doses, faster absorption, and more control over intake, making them far more effective than relying only on creatine from food sources. Additionally, creatine-based dietary supplements allow creatine to be absorbed more quickly, leading to faster creatine saturation within skeletal muscle compared to food sources (Figure 2).

It is well-established that creatine supplementation, primarily when combined with exercise training, increases measures of muscle performance, specifically muscle strength, muscle power, and the ability to repeat sprints, across a variety of populations [14]. Creatine supplementation may also aid in faster recovery from intense exercise by reducing muscle cell damage and inflammation [15]. The varied evidence supporting the efficacy of creatine supplementation as a performance-enhancing substance has made creatine a very popular ergogenic aid in recent decades [16]. It is currently the most widely used ergogenic aid in the world of sports to improve strength and physical performance in high-intensity sporting disciplines [17,18]. Despite creatine’s popularity, questions remain about optimum dose, type of administration protocol, combination with other dietary supplements, safety in special populations (vegans and women), and therapeutic interest in addressing muscular and neurological pathologies [17,19,20,21].

Creatine supplementation has been extensively studied in athletes and healthy individuals. Beyond its effects on muscle performance, creatine supplementation increases body mass by augmenting fat-free mass while reducing body fat percentage. These effects on body composition are greater when creatine supplementation is combined with resistance exercise [22]. We recommend reading prior studies that provide an in-depth analysis of creatine’s potential benefits in sports contexts, including both effects on performance and muscle mass [14,23,24]. However, the impact of creatine supplementation on specific populations with distinct physiological and dietary characteristics remains a relatively unexplored area. This is important, as previous studies have found other benefits of creatine supplementation, such as improved cognitive function [22], enhanced hydration status [25], and potential anti-aging effects to combat sarcopenia and cachexia [26,27]. Since most studies have focused on male populations, there is a gap in the scientific literature on how creatine supplementation may affect body composition and physical performance in women [28,29]. Additionally, plant-based diets are becoming increasingly popular in modern societies, driven by growing awareness of health, environmental, and ethical considerations. In these dietary patterns, where animal-based foods are excluded (i.e., vegan and vegetarians), creatine may be absent or present in limited amounts, making it essential to explore the effect of creatine supplementation as vegans and vegetarians have lower basal intramuscular creatine levels [30]. Finally, in populations with neurological conditions, creatine supplementation has shown potential benefits in improving brain function and in the management of certain diseases [31]. Given the importance of creatine in cellular energy production and its involvement in cognitive and neurological processes, understanding how supplementation with exogenous sources of creatine may have significant implications for public health and the management of certain pathologies of a muscular and neurological nature. While creatine supplementation is well-documented for enhancing athletic performance, this narrative review uniquely explores its applications beyond sports, focusing on underrepresented populations such as women, vegans, and individuals with clinical conditions. It also examines the comparative advantages of different creatine forms. By summarizing the role of creatine in supporting health and performance in these underexplored groups, this review aims to fill existing knowledge gaps and provide a comprehensive analysis of its benefits and practical applications.

## 2. Types of Creatine

Creatine is widely marketed as a dietary supplement and is available in various forms, each catering to different preferences and needs. Among these, the most prominent types include the following.

### 2.1. Creatine Monohydrate

Creatine monohydrate is a widely investigated and effective form of creatine used to increase the body’s stores of creatine, primarily in muscle tissue. Overall, creatine monohydrate supplementation is popular in fitness, sports, and some clinical settings due to its effectiveness, affordability, and high bioavailability compared to other creatine forms [5]. The most popular supplementation protocol with creatine monohydrate involves loading with 0.3 g/kg/day for 5 to 7 days, followed by a maintenance dose of 0.03 g/kg/day, typically for 4 to 6 weeks. However, loading doses are not necessary to increase intramuscular creatine stores [32]. Creatine monohydrate is a relatively safe supplement with few adverse effects reported in healthy individuals, even with long/chronic ingestion protocols [20,33]. The most common adverse effect is transient water retention in the early stages of supplementation. This water retention occurs because creatine is a hygroscopic molecule, leading to increased intracellular water content, which can enhance cellular hydration and potentially improve muscle function and recovery. For athletes and physically active individuals, this can be advantageous, as better hydration within the muscle cells may support increased strength, endurance, and resistance to fatigue. However, for some individuals, especially those monitoring their body weight or dealing with certain health conditions, the temporary increase in body mass due to water retention may be perceived as a drawback. When combined with other supplements or taken at higher than recommended doses for several months, some liver and kidney complications have been reported with creatine monohydrate supplementation [32,34]. Although further studies are needed to fully understand the potential adverse effects of prolonged creatine supplementation, supplementation with recommended doses and high-quality products is generally considered safe, particularly for healthy individuals.

### 2.2. Creatine Ethyl Ester

This type of creatine supplementation consists of creatine bound to esterified ethanol as an alternative to creatine monohydrate to optimize bioavailability or absorption [35]. The efficacy of creatine supplementation depends on the compound’s ability to effectively cross cell membranes and accumulate in target tissues, where it will be stored and utilized. As creatine enters cells primarily through active transport via the specific SLC6A8 transporter, a type of creatine that facilitates the function of this transport may be key for the effectiveness of creatine supplementation [36]. Since the capacity of the SLC6A8 transporter cannot be enhanced through creatine supplementation, some researchers have developed modified creatine molecules designed to more efficiently cross biological membranes. In 2011, the team directed by Adriano et al. designed a di-acetyl creatine ethyl ester (DAC), a compound that crosses biological membranes independently of its transporter due to its high lipophilicity [35]. Additionally, these authors also designed DAC to reduce the conversion of creatine to creatinine in an attempt to enhance the efficacy of the storage of creatine within muscles. Due to its ethanol content, the use of creatine ethyl ester is not recommended for pregnant and lactating women, children and adolescents, and individuals with hepatic or renal dysfunction [37].

### 2.3. Creatine Gluconate

Creatine gluconate consists of a creatine molecule bonded to a glucose molecule, which may enhance bioavailability and facilitate creatine entry into muscle cells. The glucose component potentially aids in increasing creatine uptake in muscles by promoting improved absorption and transport [35]. A daily intake of approximately 7.5 g of creatine gluconate supplies an effective dose of 3 g of creatine [35]. Human studies have shown that creatine supplementation combined with a high carbohydrate intake (approximately 100 g per serving) significantly increases total creatine levels in muscle tissue compared to creatine taken alone. The high carbohydrate content likely enhances creatine uptake by stimulating insulin release, which promotes creatine transport into muscle cells [38,39]. Research suggests that supplementing with 5 g of creatine monohydrate combined with 100 g of carbohydrates daily for 4 days may cause mild gastrointestinal discomfort in some individuals. This effect is likely due to the high carbohydrate load, which can increase water retention in the intestines, potentially leading to bloating or digestive upset [40]. Still, it seems safe to conclude that carbohydrate intake combined with creatine supplementation can facilitate creatine transport, although the optimal carbohydrate amount and specific creatine forms for maximizing creatine uptake are still under investigation. Research on the effectiveness of creatine gluconate specifically, and the combination of creatine with carbohydrates to enhance creatine uptake, warrants further investigation.

### 2.4. Creatine Citrate

Creatine citrate is a form of creatine in which creatine molecules are bonded with citric acid. This modification aims to enhance certain properties of the molecule, such as solubility and potential absorption, compared to traditional creatine monohydrate. Normally, one creatine molecule is bound to one or more citric acid molecules, creating a compound that is somewhat more water-soluble than creatine monohydrate. However, the ratio of the composition in which the creatine/citrate formula is presented can also be given in different ratios (1:1, 2:1, or 3:1). Dicreatine citrate (2:1) is more water-soluble than creatine and dissociates into creatine and citrate in solution, reducing the intestinal discomfort that creatine in other forms can cause. Some creatine is released as creatine monohydrate, while some is formed as creatinine [41]. The bioavailability of creatine in the form of creatine citrate has been estimated to be similar to that reported for creatine monohydrate.

### 2.5. Creatine Magnesium Chelate

Creatine magnesium chelate is a form of creatine bonded to magnesium, designed to improve the stability, absorption, and effectiveness of creatine supplementation [42]. Magnesium is a critical mineral involved in ATP production, and both creatine and magnesium play overlapping roles in muscle bioenergetics, as the dephosphorylation of PCr by creatine kinase requires magnesium as a cofactor. This makes the combination of creatine and magnesium potentially synergistic for energy metabolism and muscle performance [43]. Studies suggest that creatine magnesium chelate may enhance creatine uptake and bioavailability compared to creatine monohydrate, potentially reducing the typical water retention associated with creatine use [42]. However, further studies are needed to confirm its superiority over other creatine forms and its long-term safety and efficacy.

Collectively, creatine monohydrate is the form most commonly used in the vast majority of scientific research and therefore has the most scientific evidence [14]. Therefore, from a practical perspective, this form of creatine should be preferential when seeking creatine supplementation benefits in athletes or clinical populations. Alternative forms of creatine offer promising approaches to enhance creatine supplementation; however, they require further scientific investigation before they can be recommended as preferred forms over creatine monohydrate. Many sports supplements on the market contain creatine blended with other nutritional components to create a cocktail of ergogenic and supportive ingredients. These commonly include glucose/carbohydrates, proteins, vitamins, caffeine, and herbal extracts, all aimed at enhancing performance and recovery. The scientific literature has not demonstrated that cocktails of substances combined with creatine increase the effects of acute creatine intake. Furthermore, no consistent evidence or studies assessing the health-related safety of these cocktails have been found to date [32,44]. Therefore, the intake of such a combination of substances as a strategy to optimize the ergogenic effects of creatine also requires investigation.

## 3. Physiological Effect of Creatine

Approximately 95% of the body’s creatine is stored in skeletal muscle, where it is highly concentrated, reaching levels of around 130 mmol per kilogram of dry muscle mass. Within muscle cells, about 60% of creatine is stored as phosphocreatine, while the remaining 40% exists as free creatine. During the transition from rest to exercise, phosphocreatine acts as an immediate energy reservoir, rapidly donating phosphate to re-phosphorylate ADP to ATP. This provides a quick supply of energy for muscle contractions, sustaining high-intensity activity for the first 6–8 s before anaerobic glycolysis becomes the primary energy source [45]. Temporary energy buffering is also linked to the activation of mitochondrial respiration, which forms a creatine–phosphocreatine shuttle called the “spatial energy buffer” [46]. In addition to creatine’s role in rapid energy production during high-intensity exercise, it has been shown to exert other physiological effects, including upregulating the expression of various genes. Some of these effects may be related to cellular responses to inflammation, as demonstrated in animal models [47]. Some of these cellular effects may be responsible for studies demonstrating the beneficial effects of creatine supplementation in various animal and human models of neurological diseases [48,49,50].

Interest in creatine supplementation for sport and physiology studies arose in 1992 when Harris et al. [51] demonstrated that after 5 days of oral administration of creatine monohydrate (20 g/day), total muscle creatine and phosphocreatine increased by approximately 15–20%. Subsequent investigations demonstrated similar increases in creatine and phosphocreatine after 30 days of low-dose creatine administration (3 g/day) and after a loading dose (20 g/day for 6 days) and 2 g/day for 1 month [52,53,54]. Elevated creatine levels in muscle slowly returned to normal after 5–8 weeks following cessation of supplementation [55]. Today, creatine supplementation is not on the World Anti-Doping Agency’s (WADA) list of prohibited substances, as the evidence of the benefits of creatine supplementation has not been accompanied by evidence of this substance increasing the risk of health problems in athletes.

Intramuscular creatine levels vary significantly between individuals, influenced by factors such as diet, sex, age, and genetics. Vegetarians, for example, tend to have lower muscle creatine levels compared to individuals who consume meat or fish [56]. Additionally, women generally have lower muscle creatine levels than men, and levels may decline with age. Assessing muscle creatine concentration requires muscle biopsy, which allows for precise measurement and can highlight inter-individual differences. These intramuscular creatine variations may stem from genetic factors, such as differences in creatine transport capacity, as well as dietary habits, particularly meat and fish intake. Future studies should take these dietary, genetic, sex, and age-related factors into account to deepen understanding of creatine variability across populations.

## 4. Creatine and Physical Exercise

Creatine is one of the most used ergogenic nutritional aids by athletes of all levels. Studies have shown that the effective dose of creatine supplementation consists of a loading dose of 0.3 g/kg/day for 5–7 days, followed by a maintenance dose of 0.03 g/kg/day for 4–6 weeks thereafter [32,44], which helps to increase intramuscular PCr concentrations by promoting phosphagen replenishment [57]. Additionally, creatine supplementation without an initial loading phase has also been shown to effectively produce PCr accumulation in skeletal muscle. This physiological mechanism helps explain the improvements in high-intensity exercise performance and the enhanced training adaptations commonly associated with creatine supplementation [38,58]. It has also been shown that creatine supplementation can increase the rate of glycogen replenishment, which would be of great interest in athletes who perform prolonged submaximal efforts (65–75% of the maximum oxygen consumption rate (VO_2max_)) [59] or perform repeated high-intensity exercise [60]. Many sports involve a combination of high-intensity actions—whether isolated or repeated—which rely heavily on optimal anaerobic metabolism, alongside periods of low-intensity activity that require efficient aerobic metabolism. Such sports may benefit from creatine supplementation, as it supports both quick energy release during intense efforts and recovery during lower-intensity phases [61]. Conversely, current evidence indicates that creatine supplementation may be ineffective in endurance performance [62].

### Sports Physiology and Creatine

The importance of phosphocreatine hydrolysis as the primary energy source during exercise varies with exercise intensity, duration, and frequency. During short and maximal-intensity sprints (e.g., 6 s in duration), power output exceeds approximately 250% of maximal oxygen consumption, with roughly 50% of energy coming from phosphocreatine hydrolysis and the remaining 50% primarily derived from cytosolic glycolysis. These energy pathways work in tandem to support the high-intensity demands of short-duration, maximal efforts [63]. On the other hand, during a 30 s sprint at 200% of maximal oxygen consumption, cytosolic glycolysis contributes ∼55% of the total ATP requirement, with PCr hydrolysis contributing only ∼25% and oxidative phosphorylation the remaining 50% of energy [64,65]. When exercise duration exceeds 2 s at high intensities, the concentration of phosphocreatine drops significantly [66]. If high exercise intensity is sustained for prolonged periods, phosphocreatine levels will deplete, lactate production will rise, and muscle fatigue will ensue, ultimately impairing the ability to maintain intense exercise. Creatine supplementation helps increase phosphocreatine stores, enabling athletes to sustain high-intensity efforts for longer durations before fatigue sets in [14,54]. An increase in PCr concentration provides a greater energy reserve for high-intensity efforts [33], supporting enhanced performance in sports that involve short-duration, intermittent activities with a predominantly anaerobic component. This demonstrates that creatine supplementation can improve performance in exercises that require rapid energy release and recovery, particularly in anaerobic, high-intensity sports [67,68].

On the other hand, creatine supplementation in aerobic sports shows a low ergogenic effect. Different studies show that creatine supplementation does not increase maximal oxygen consumption, submaximal oxygen consumption, or time trial performance [14,16], due to the fact that the percentage of the phosphogenic energy pathway is low [44], with the energy pathways of fat and carbohydrate oxidation being more in demand. Although it has been suggested that the water retention associated with creatine supplementation could enhance endurance performance, this mechanism has been shown to be ineffective in most studies examining creatine supplementation in endurance athletes [62]. Another justification for the absence of an ergogenic effect in sports with a high aerobic component is the fact that the increase in the body mass of the subjects after treatment with creatine would lead to a possible neutralization of the beneficial effects, with body mass being of great relevance in these sports for optimal efficiency [69].

## 5. Potential Adverse Effects of Creatine Supplementation

Creatine supplementation, especially in loading protocols where large amounts are ingested at the beginning of the supplementation protocol, can markedly reduce normal creatine synthesis in the body. However, after a short period of cessation of creatine supplementation, creatine synthesis within the body returns to baseline values within 4–6 weeks [70]. Despite numerous studies on the ergogenic effects of creatine supplementation, there is limited information on potential adverse effects, as most investigations have not reported any side effects conveyed by participants [33]. There are limited reports suggesting that creatine supplementation may have protective effects against cardiac, muscular, and neurological diseases. While gastrointestinal disturbances and muscle cramps have occasionally been reported in healthy individuals, these effects are generally anecdotal. Hepatic and renal dysfunction have also been suggested based on small changes in organ function markers and isolated case reports. However, there is a lack of well-controlled studies investigating the potential adverse effects of exogenous creatine supplementation [33]. To date, no adverse events related to renal function have been observed with creatine supplementation, even at doses of up to 5 g per day over prolonged periods (typically 6 months or longer), indicating that such doses are considered safe for healthy individuals. The scientific evidence is inconclusive or insufficient to categorize creatine supplementation as a health risk [14]. From a practical standpoint, although there is no evidence to suggest health risks associated with chronic creatine supplementation, it may be beneficial to discontinue supplementation at certain times of the year. This strategy could optimize creatine’s effects during periods of higher training volume, intensity, or important competition phases. Taking breaks from creatine supplementation allows the body to reset and may enhance its efficacy during key performance periods. This approach has been suggested by recent studies focusing on periodized creatine use to maximize athletic performance and recovery [67,71].

### 5.1. Fluid Retention During Supplementation with Creatine Monohydrate

Creatine monohydrate supplementation has been shown to increase body mass by 1–3 kg after short-term use (5–7 days) [72,73]. Several studies have shown a significant reduction in urine output during the first 3 days of the creatine-loading period [44,55]. This water retention is related to an osmotic load caused by creatine retention [70] and explains the rapid weight gain experienced by those ingesting creatine [74]. This increase in bodily fluid and total intracellular fluid following creatine supplementation is also observed in women, with no significant differences across the menstrual cycle [75]. These changes are not observed in long-term studies (4–6 weeks), suggesting that the increase in body fluid is transient [76,77]. Water retention associated with creatine supplementation can be advantageous for certain sports, particularly those that emphasize aesthetic factors, such as bodybuilding, physique competitions, or fitness modeling. This retention can contribute to increases in non-sarcomere muscle mass, enhancing the appearance of muscle fullness and size, which are critical in these disciplines. Overall, creatine supplementation may promote rapid changes in body composition, enhance muscle cell volumization, and potentially improve recovery following intense training. Its ability to increase water retention helps maintain fluid balance in muscle cells, which can reduce muscle damage and inflammation post-exercise, aiding in faster recovery. While water retention from creatine supplementation can enhance muscle hydration and recovery, it may also have drawbacks for some athletes. The added weight from increased water retention could negatively affect performance in sports requiring agility, speed, or weight class considerations, as the additional mass may impair movement efficiency or exceed weight limits for certain competitions.

### 5.2. Renal Function and Creatine Monohydrate Supplementation

Supplementation with creatine monohydrate has been shown to increase creatinine levels, a by-product of creatine metabolism that is typically excreted by the kidneys. However, current research suggests that this acute rise in creatinine concentration does not indicate any harmful effects on kidney function or structural integrity, particularly in healthy individuals. The increase in creatinine is primarily due to elevated creatine stores in the muscles, which leads to higher breakdown rates. Importantly, studies have not found evidence linking creatine supplementation to long-term damage to the nephron, the kidney’s basic functional unit. As such, there is no cause for concern regarding kidney function in individuals with normal renal health, though further investigation is warranted in populations with pre-existing kidney conditions. In fact, several studies have demonstrated that chronic creatine supplementation, even over extended periods such as 5 years, does not adversely affect kidney function in healthy athletes [78,79]. Research has consistently shown that, in individuals with normal renal function, long-term creatine use does not lead to any significant changes in key kidney markers, such as glomerular filtration rate (GFR) or serum creatinine levels, beyond the temporary increase seen during supplementation [80]. Additionally, creatine supplementation has been shown to provide potential protective effects in certain clinical contexts, such as improving muscle mass and energy metabolism in hemodialysis patients [81], although these applications require more robust clinical evidence. Patients with chronic kidney disease (CKD) often experience debilitating symptoms such as fatigue, muscle weakness, muscle wasting, and sarcopenia. These symptoms likely contribute to the diminished quality of life and increased risk of premature mortality commonly associated with CKD. Given creatine’s critical role in muscle energy metabolism and overall cellular function, it is essential to explore whether increasing dietary creatine intake or supplementing with creatine could mitigate these effects in CKD patients. Further research is urgently needed to evaluate the safety, efficacy, and practical application of creatine supplementation in this population, particularly under medical supervision, to determine its potential as a therapeutic intervention [82].

Collectively, while creatine supplementation can lead to a transient rise in creatinine due to increased muscle mass and creatine turnover, it does not impair kidney function or cause structural damage to the nephron. However, it is important to note that these findings generally apply to healthy individuals, and further research is necessary to assess the effects of long-term creatine supplementation in populations with pre-existing kidney conditions or other underlying health concerns.

## 6. Creatine Supplementation Beyond Athletics

As exposed before, creatine supplementation is widely recognized for enhancing athletic performance, particularly in high-intensity and strength-based sports, and for promoting faster body composition changes when combined with exercise programs. However, its potential benefits extend beyond athletics to underexplored populations, including women (both athletes and physically active individuals), vegans, and individuals with clinical conditions. Women may experience unique benefits from creatine supplementation, such as improved energy metabolism during the menstrual cycle. Vegans, who lack dietary creatine due to the absence of animal-based foods, may benefit more significantly from supplementation as it restores depleted muscle creatine levels. In clinical populations, creatine holds promise in mitigating muscle wasting, supporting neuroprotection, and improving cardiovascular and metabolic health through its critical role in cellular energy metabolism. Despite these potential advantages, these areas remain comparatively under-researched relative to the extensive focus on creatine’s benefits in sports.

## 7. Creatine Supplementation in Women

In women, a higher proportion of PCr has been identified at the intramuscular level [83]. However, the endogenous creatine synthesis rate is around 70–80% compared to that reported in men [84]. Similarly, dietary creatine intake is lower in women, suggesting an interest in creatine supplementation as a strategy to preserve PCr stores in female athletes [84]. Furthermore, the ergogenic effects of creatine may vary according to the metabolic changes associated with the different phases of the menstrual cycle. Thus, it has been reported that creatine supplementation during the luteal phase reduces fatigue after a sprint test, whereas no significant changes were reported during the follicular phase [85]. Interestingly, creatine supplementation could be useful to counteract some of the physiological changes linked to the menopausal process, such as the alteration of bone tissue [86]. However, these benefits appear to be dependent on coexistence with physical exercise, as no improvements in bone health are reported with creatine administration alone [87].

For women, creatine supplementation can provide significant benefits for improving exercise performance, muscle strength, and recovery [88]. The recommended dosing for women is similar to that of men, typically 3–5 g per day of creatine monohydrate. While some women may choose to follow a loading phase (20 g per day for 5–7 days), research suggests that a lower, consistent dose (3–5 g per day) can be equally effective over time, without the need for an initial high-dose phase [14]. The timing of creatine supplementation can vary, but taking it post-exercise may be beneficial, as it can aid in muscle recovery and enhance the replenishment of phosphocreatine stores [69]. Regarding the menstrual cycle, its exact effects on creatine efficacy are still not fully understood, and more research is needed. However, since general creatine supplementation guidelines are effective in maintaining creatine stores within the muscle, adjusting intake protocols throughout different phases of the menstrual cycle appears unnecessary. Most research indicates that standard creatine supplementation provides consistent benefits across the menstrual cycle, with no evidence suggesting that varying intake timing or dosage significantly enhances the effects in relation to hormonal fluctuations. Women should also consider their individual responses to creatine supplementation. Some may experience weight gain due to water retention, especially during the initial stages of supplementation, which is generally more noticeable when following a loading phase. In summary, creatine monohydrate supplementation is safe and effective for women. While further research is needed to refine protocols specific to women, the consistent and personalized use of creatine remains a valuable tool for enhancing performance and recovery. Although evidence suggests a possible advantage during the luteal phase, there is currently no conclusive recommendation on specific cycle timing for optimal creatine efficacy in women.

## 8. Creatine Supplementation for Vegetarians/Vegans

Creatine is naturally found in animal-based foods such as meat, fish, and poultry, which are the primary dietary sources of this compound. As a result, athletes who follow vegetarian or vegan diets generally consume lower levels of creatine compared to those who include animal products in their diets [56]. Dietary intake of creatine is substantially lower in individuals following a vegetarian diet, as plant-based foods contain negligible amounts of this compound. While eggs and dairy products provide small amounts of creatine, the levels are much lower than those found in meat, fish, or poultry. In vegan diets, there are virtually no natural dietary sources of creatine, as plant foods do not contain significant amounts of this compound [89]. In vegetarians, the amount of creatine is lower in serum, plasma, red blood cells, and muscle; however, no differences have been observed in the brain compared to omnivores [30,90]. In addition, vitamin B12 deficiency, which is common in non-supplemented vegans, can impair the synthesis of methionine, a critical precursor for creatine biosynthesis. Since methionine is involved in the conversion of guanidinoacetate to creatine, a lack of adequate vitamin B12 may result in reduced endogenous creatine production [91]. This contributes to lower creatine stores in the muscles, potentially affecting performance in activities reliant on high-intensity energy production in vegans and vegetarians. Therefore, creatine supplementation appears to be a useful approach to offset some of the nutritional concerns associated with vegetarian and vegan diets.

Given that creatine and PCr concentrations are typically lower in the muscles of vegetarians and vegans, creatine supplementation may offer significant benefits by increasing these concentrations and potentially improving exercise performance and recovery. Research suggests that vegetarians and vegans, due to their lower baseline levels of creatine, may experience a more pronounced improvement in muscle creatine stores from supplementation compared to omnivores [92]. Strikingly, a possible super-compensation phenomenon associated with creatine supplementation has been described in a vegetarian population. Burke et al. [30] reported a greater increase in muscle PCr levels after creatine supplementation among vegetarian participants compared to omnivores, despite the latter starting with higher baseline levels. However, other studies have not reported the superiority of creatine supplementation in the vegetarian population with respect to individuals with omnivore diets. It should be noted that although creatine is mainly found in animal products, the creatine in most supplements is synthesized from sarcosine and cyanamide [93]. Therefore, it does not contain any animal by-products and is ’vegan-friendly’. The only caution is that vegans should avoid creatine supplements supplied in capsule form, as these are often derived from gelatin and therefore may contain animal by-products. Even so, there are still limitations in the reporting of the ergogenic effects of creatine in vegan subjects that need to be enhanced in future studies [94].

For practical recommendations, for vegans and vegetarians seeking to enhance their high-intensity exercise performance or recovery, creatine supplementation offers a promising solution, especially given their typically lower baseline muscle creatine levels. While creatine supplementation may be effective in improving muscle creatine stores, it is important to ensure proper dosing, as the individual responses to this substance can vary significantly. In this regard, the creatine dosing recommendations outlined in previous sections of this paper are equally applicable to vegans and vegetarians. Despite these individuals showing a potentially greater response to creatine supplementation due to their lower baseline creatine stores, research does not support the use of higher doses for optimal results. It is also recommended to cycle creatine supplementation, particularly during high-intensity training phases or competition periods, to optimize its effects and prevent possible long-term tolerance. Although more research is needed on its effects specifically in vegan populations, creatine remains a safe and beneficial ergogenic aid for vegetarians and vegans. Following the established creatine supplementation protocols for the general population is recommended for these populations.

## 9. Therapeutic Potential of Creatine Supplementation in Diseases

Considering that the primary action of creatine supplementation occurs at the muscular level, various studies have investigated its potential effects on myopathies [95]. Low creatine and PCr levels in individuals with various types of muscular dystrophy have been shown to increase with creatine supplementation, leading to notable improvements in muscle strength [96]. Research demonstrates that a creatine supplementation protocol can significantly enhance muscle function in these populations, likely due to increased energy availability and improved muscle cell metabolism [69,97]. However, long-term studies are needed to corroborate the efficacy and safety of creatine supplementation, as well as an assessment for each specific type of myopathy.

The presence of a specific form of creatine kinase in the brain suggests that creatine plays a vital role in brain energy metabolism. Several studies have tested this hypothesis and found that creatine supplementation can lead to improvements in cognitive function, such as memory, attention, and mental fatigue [98,99]. These cognitive benefits are particularly noticeable under conditions of mental or physical stress, where creatine supplementation appears to support enhanced cognitive processing by increasing available energy within brain cells [100]. Creatine supplementation may be beneficial not only in pathological conditions but also in healthy individuals to enhance or maintain normal cognitive function [101]. Furthermore, these beneficial effects on cognitive function appear to be more pronounced in vegetarians, due to lower levels of phosphocreatine [102]. However, these results contrast with those found in previous long-term studies, which have found no evidence of improvements in cognitive function following creatine supplementation of either 10 g/day or 20 g/day compared to placebo for 6 weeks [103]. Future research on creatine supplementation in cognitive function faces several challenges, including the need for long-term clinical trials to determine efficacy and safety, the identification of optimal dosing strategies, and a deeper understanding of the underlying mechanisms by which creatine impacts cognitive function.

Huntington’s disease is a neurodegenerative condition characterized by impaired cognitive function, concomitant with disruption of energy metabolism and decreased phosphocreatine regeneration. The study by Hersch et al. [104] demonstrated that supplementation with 8 g/day of creatine for 16 weeks in Huntington’s disease patients was able to induce a decrease in levels of 8-hydroxy-2′-deoxyguanosine (8OH2’dG), a biomarker of oxidative DNA damage. In contrast, no changes in disease progression after creatine supplementation have been reported in other studies [105]. Similarly, a reduction in dopaminergic therapy following co-administration of creatine has been reported in Parkinson’s disease subjects [106], but no clinical benefits have been found in long-term studies based on validated scales for assessing disease progression [107].

Creatine supplementation has also garnered interest for its potential therapeutic benefits in the context of cardiovascular disease. Although the primary mechanism of creatine is related to energy production within muscle cells, recent research suggests that it may also offer cardiovascular protection through several mechanisms, including improving endothelial function, reducing oxidative stress, and enhancing blood flow [108,109]. Creatine supplementation has been shown to enhance endothelial function by improving nitric oxide production, which promotes vasodilation. This effect could lead to improved blood flow and reduced vascular resistance, potentially benefiting individuals with hypertension or other cardiovascular risk factors. Oxidative stress is a significant contributor to the development of cardiovascular diseases, as it accelerates the process of atherosclerosis and inflammation. Creatine’s antioxidant properties have been demonstrated in animal models, where it was shown to reduce oxidative stress markers [109]. In addition to its role in energy metabolism, creatine appears to act as a buffer against the harmful effects of reactive oxygen species in cardiovascular tissues, potentially slowing the progression of cardiovascular disease [110], although this is a topic that deserves further investigation as not all evidence shows benefits of higher intracellular levels of creatine [111]. Creatine supplementation has also been explored as a potential intervention for improving heart function in individuals with heart failure. In heart failure, creatine and phosphocreatine decrease because of the decreased expression of the creatine transporter, and because phosphocreatine degrades to prevent adenosine triphosphate (ATP) exhaustion. Research indicates that creatine can enhance myocardial energy availability, improve contractility, and increase cardiac output in heart failure patients [110,112]. Some studies have shown that creatine can help improve exercise tolerance and reduce fatigue in these individuals, possibly through its effects on mitochondrial function and ATP regeneration in cardiac muscle cells [7]. Finally, some studies suggest that creatine supplementation can aid in the rehabilitation process by supporting muscle energy stores, reducing muscle wasting, and enhancing overall physical function during recovery [113].

Collectively, evidence supports that creatine supplementation offers significant potential in the management of both cognitive diseases and myopathologies, with numerous studies supporting its benefits. For cognitive diseases, creatine enhances brain function and offers neuroprotection, while for myopathologies, it improves muscle function and strength. There are also promising benefits of creatine supplementation for prevention or as a coadjutant in the treatment of cardiovascular diseases. However, further research is needed to better define optimal dosages, long-term effects, and specific patient groups that would benefit most from supplementation. Regular monitoring, particularly of renal function, is advisable for individuals with existing conditions.

## 10. Conclusions

Creatine can be endogenously synthesized from amino acids or absorbed from foods such as meat and fish. However, the intake of dietary supplements containing creatine augments both serum creatine concentration and muscle creatine concentration, as the rates of endogenous creatine synthesis (1 g/day) or ingestion through foods (1–2 g/day) are very limited in comparison to the doses habitually ingested through dietary supplements (from 5 to 20 g/day depending on the loading protocol). Although research has shown no significant differences in the effectiveness of continuous daily intake (e.g., 3–5 g per day) versus loading protocols (20 g/day for 5–7 days followed by a maintenance dose), adopting a continuous daily creatine supplementation protocol is a more practical and convenient approach compared to traditional loading protocols. Daily intake eliminates the need for high initial dosages and minimizes the potential gastrointestinal discomfort that is sometimes associated with loading protocols. Creatine monohydrate is the most recommended form of creatine supplementation due to its high bioavailability, ability to increase creatine levels in muscles and other tissues, proven safety record when used appropriately, and generally lower cost compared to other forms, such as creatine ethyl ester and creatine hydrochloride.

Creatine supplementation effectively increases intramuscular creatine and phosphocreatine, which provides an increase in energy stores for high-intensity work. Additionally, due to creatine’s hygroscopic nature, creatine supplementation causes water retention. While this effect can be advantageous in certain situations, such as promoting cellular hydration and muscle volumization during intense physical activity, it may also be perceived as a side effect by some individuals, particularly those concerned about temporary weight gain. Creatine supplementation is safe in long- and short-term protocols for healthy men and women, as well as for young and older people. For women, a continuous daily protocol can be especially beneficial, as it avoids the fluctuations in creatine supplementation that might coincide with hormonal changes throughout the menstrual cycle. This consistency supports stable energy metabolism and performance benefits, particularly during phases of higher fatigue. Creatine supplementation provides more significant increases in intramuscular creatine in vegans than in omnivores, due to lower initial levels of creatine stores. For vegans, it is important to note that most commercially available creatine supplements, including creatine monohydrate, are synthesized chemically and do not come from animal sources. This makes creatine supplementation a “vegan-friendly” option. However, vegans should ensure that the creatine product they choose does not include non-vegan additives, such as gelatin in capsule formulations. For individuals using creatine supplementation to support cognitive function or manage clinical conditions, it is recommended to adhere to a consistent daily dosage of 3–5 g per day of creatine monohydrate, as this dose has been widely studied and shown to be effective in improving energy metabolism in the brain. This steady supplementation approach ensures sufficient creatine availability to support neuroprotection, cognitive enhancement, or energy demands associated with clinical conditions such as neurodegenerative diseases (e.g., Parkinson’s and Huntington’s) or traumatic brain injuries. Before initiating creatine supplementation, women, vegans, or individuals with clinical conditions are strongly advised to consult with dietitians or healthcare professionals to assess the suitability of creatine for their specific needs. The supplementation protocol, including dosage and duration, should be tailored to the individual’s characteristics, health status, and dietary patterns. This is particularly important for those who are on medications, as professional guidance can help prevent potential interactions and ensure the safe and effective use of creatine. Lastly, creatine supplementation should be combined with a balanced diet and, if applicable, regular physical activity to optimize overall health outcomes. Monitoring progress and any side effects with periodic evaluations can further ensure that supplementation is tailored to the individual’s needs.

**Figure 3:**
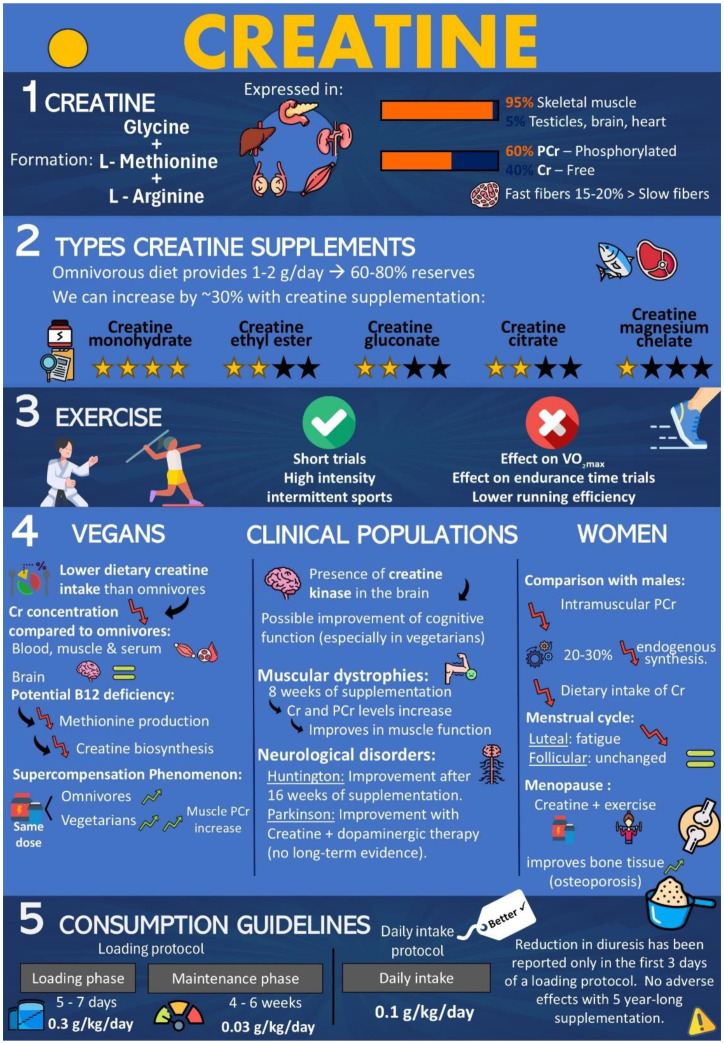
Infographic of the benefits of creatine supplementation in women, vegans, and clinical populations.

In summary, creatine monohydrate supplementation can enhance physical performance, cognitive function, and overall health in women, vegans, and clinical populations. A summary of the topics afforded in this review can be depicted in the following infographic (Figure 3).
